# Self-healing hydrogel as an injectable implant: translation in brain diseases

**DOI:** 10.1186/s12929-023-00939-x

**Published:** 2023-06-20

**Authors:** Junpeng Xu, Shan-hui Hsu

**Affiliations:** 1 Institute of Polymer Science and Engineering, National Taiwan University, No. 1, Sec. 4 Roosevelt Road, Taipei, 106319 Taiwan, Republic of China; 2 Institute of Cellular and System Medicine, National Health Research Institutes, No. 35 Keyan Road, Miaoli, 350401 Taiwan, Republic of China

**Keywords:** Self-healing hydrogel, Injectable implant, Neural tissue engineering, Translation medicine, Stroke, Neurodegenerative disease, Traumatic brain injury

## Abstract

Tissue engineering biomaterials are aimed to mimic natural tissue and promote new tissue formation for the treatment of impaired or diseased tissues. Highly porous biomaterial scaffolds are often used to carry cells or drugs to regenerate tissue-like structures. Meanwhile, self-healing hydrogel as a category of smart soft hydrogel with the ability to automatically repair its own structure after damage has been developed for various applications through designs of dynamic crosslinking networks. Due to flexibility, biocompatibility, and ease of functionalization, self-healing hydrogel has great potential in regenerative medicine, especially in restoring the structure and function of impaired neural tissue. Recent researchers have developed self-healing hydrogel as drug/cell carriers or tissue support matrices for targeted injection via minimally invasive surgery, which has become a promising strategy in treating brain diseases. In this review, the development history of self-healing hydrogel for biomedical applications and the design strategies according to different crosslinking (gel formation) mechanisms are summarized. The current therapeutic progress of self-healing hydrogels for brain diseases is described as well, with an emphasis on the potential therapeutic applications validated by in vivo experiments. The most recent aspect as well as the design rationale of self-healing hydrogel for different brain diseases is also addressed.

## Background

The increasing demand for biomaterials in recent decades to regenerate the impaired tissues has driven the new development in tissue engineering [1, 2]. Biomaterials for tissue engineering need to have the capability to mimic and stimulate natural tissues in order for the treatment of damage and diseased tissues [3, 4]. So far, most tissue engineering therapies have relied on the delivery of stem cells by native-like and highly porous biomaterials, i.e., tissue-engineered scaffolds [5–7]. The scaffolds allow the embedded cells to diffuse and reconstruct into tissue-like structures at the target site, while allows adequate nutrient and waste interchange with the surrounding environment [8, 9]. Among numerous scaffolds applied in tissue engineering, hydrogel is one of the most promising cell carriers.

Hydrogel can well mimic extracellular matrix (ECM) because of its three-dimensional network structure with high water content [10]. Since the networks of hydrogels are prepared through various chemical or physical crosslinking mechanisms, they can provide a broad range of functions to the hydrogels, such as elasticity, biocompatibility, and biodegradability, to meet the requirement for different tissues [11]. Conventional hydrogel is normally not sensitive to changes in the environment, such as temperature or pH. When the hydrogel is subjected to external force, it would crack and fail to repair itself, which may make the hydrogel counterproductive in tissue repair and thus limit its applications [12]. Hydrogel with a permanent crosslinking network may affect the regular growth and reproduction of cells [13]. Preparing a hydrogel using dynamic crosslinking strategies can endow the hydrogel with self-healing properties, which has been an attractive research topic in recent years [14, 15].

The concept of self-healing materials was inspired by the phenomenon of self-healing in the human body, such as the ability of the skin to recover its original shape and function after injury [16, 17]. Self-healing hydrogel is a category of smart hydrogels with the inherent ability to repair damage automatically without external intervention [18, 19]. The self-healing properties of hydrogels are achieved through a crosslinked network of dynamic/reversible bonding. Because of the flexibility, biocompatibility, and ease of functionalization, many self-healing hydrogels with unique properties and prolonged longevity have been developed by designing dynamic crosslinking networks for various biomedical applications [20]. Self-healing hydrogels are generally applied in vivo by injection. Many studies have shown that self-healing hydrogels have no apparent damage to the organism after injection into the target site, and that the structural and functional integrity of the target tissue can be maintained after injection [21, 22]. Therefore, self-healing hydrogels show great potential in regenerative medicine and deserve more translational studies.

Due to demographic changes, a further increase in the incidence of brain diseases, e.g., neurodegenerative and cerebrovascular disorders, can be expected in the modern society. Restoring the structure and function of impaired neural tissues is one of the most difficult challenges that human beings must face because the injured neural system has a limited capacity for self-healing, especially the central nervous system (CNS). Although a few studies have developed effective drugs that exhibit positive effects on some brain diseases, there are still problems with low therapeutic efficiency, drug resistance, and inability to cross the blood–brain barrier [23]. Cell therapy also shows certain efficacy in neurological disorders, but the treatment outcome is highly dependent on the selected carrier [24].

Using self-healing hydrogels as drug/cell carriers for targeted injection via minimally invasive surgery has been developed by recent researchers and has become a promising biomedical strategy. Considerable progress has been achieved in various animal models, such as the rat intracerebral hemorrhage (ICH) model and the rat Parkinson's disease (PD) model [21, 25]. In addition, the use of multifunctional self-healing hydrogels alone has also been demonstrated to possess therapeutic effects in PD through animal studies very recently [22]. Therefore, self-healing hydrogels can be potentially effective in either role they play, i.e., carriers or scaffolds, to treat the brain diseases, but unfortunately there is no practical translation of self-healing hydrogels from animal studies to clinical trials. In this review, the development history of self-healing hydrogels for biomedical applications and the design strategies according to different crosslinking mechanisms are summarized. The current therapeutic progress of self-healing hydrogels for brain diseases is also described, with an emphasis on the potential therapeutic applications validated by in vivo experiments. Research trends indicate that self-healing hydrogels with reversible crosslinking networks may have a broad and uprising scope in translation medicine for utilization in clinics in the future.

## Development of self-healing hydrogel for biomedical purposes

Self-healing hydrogels, as a very important class of soft materials, have emerged during the last decade. Self-healing hydrogels in the biomedical field with in vitro cell experiments to confirm their biocompatibility were first reported in 2009, in which Jin et al. [26] developed a physically crosslinked self-healing hydrogel composed of chitosan, poly(ethylene oxide), and silica nanoparticles. However, the verification of biocompatibility was performed by preparing hydrogels as thin films and seeding NIH 3T3 cells on the surface instead of growing cells in the three-dimensional (3D) environment of the hydrogels. In the same year, Foo et al. [27] generated a two protein-engineered physical hydrogel with self-healing property assembled via molecular recognition for the encapsulation of neural stem cells (NSCs). These literatures revealed that earlier self-healing hydrogels for biomedical applications were prepared based on hydrogen bonding and peptide assembly. Afterwards, more versatile self-healing hydrogels have been developed for biomedical applications over the period in three stages, i.e., 2011–2013, 2014–2015, and after 2015.

In the first stage of development from 2011 to 2013, self-healing hydrogels generated by chemical crosslinking mechanisms, besides hydrogen bonding and peptide assembly, have been successfully reported and demonstrated preliminary effects in cell-containing injections and various animal experiments. Zhang et al. [28] successfully synthesized difunctionalized polyethylene glycol (DFPEG) and fabricated self-healing hydrogels in combination with chitosan for drug release in 2011, introducing the Schiff reaction as a dynamic chemical crosslinking mechanism to the general public and becoming popular even until today. After that, Wei’s team successively prepared magnetically responsive self-healing hydrogels by adding iron oxide to the chitosan/DFPEG system in the following year as well as validated the cytotoxicity of DFPEG [29]. They also showed cell survival in the 3D environment of glycol chitosan (GC)/DFPEG hydrogel and proposed the possibility of injection for the self-healing hydrogel system based on Schiff reaction [30]. A hydrogel based on graphene and acrylic network with an electrical conductivity of 0.67 × 10^−4^ Siemens/cm (0.067 mS/cm) possessed thermal/near-infrared laser triggered self-healing properties was also reported in 2012 [31]. The latter work brought multiple functions, such as conductivity and stimulus responsiveness, to a biomedical self-healing hydrogel for the first time. Meanwhile, Burdick’s team focused on the development of injectable shear-thinning hydrogels with self-healing properties based on the Dock-and-Lock (DnL) mechanism by 2014 and demonstrated that cell-loaded hydrogels well maintained the cellular viability after injection [32, 33]. Notably, a protein self-assembled hydrogel [34] and a supramolecular self-assembled hydrogel [35] with injectable and self-healing properties have been evaluated in vivo through animal studies for various functionalities, including cell delivery, in situ gelation, drug delivery, and myocardial tissue remodeling and repair. Among these, Bastings et al. [35] published the first study on the application of self-healing hydrogel to a large animal (pig) model.

The second phase, i.e., 2014 to 2015, was a period of rapid development, with a mushrooming of self-healing hydrogels for biomedical purposes with multiple crosslinking mechanisms being reported, including peptide assembly [36–38], host–guest interaction [39–41], hydrogen bonding [42, 43], micellar stacking [44], Schiff base linkages (imine bond [45, 46] and hydrazone bond [47–49]), metal coordination bonding [48], metal-thiolate/disulfide bonding [50], Diels–Alder click reaction [49], and zwitterionic fusion [51]. Among these crosslinking mechanisms, the non-covalent crosslinking mechanism especially the host–guest interaction was still dominant in the preparation of biomedical self-healing hydrogels, compared to the covalent bonding. Biomedical double-network hydrogels with self-healing properties were also reported in this period, such as imine bond combined with hydrazone bond [52], peptide assembly combined with hydrogen bonding [53], host–guest interaction combined with microcellular stacking [44], and hydrazone bond combined with Diels–Alder click reaction [49]. Meanwhile, 3D bioprinting using self-healing hydrogel containing living cells as “bioink” was developed and it provided both the direct printing and “gel in gel” printing as ideas for fabrication, which had become a new direction for the application of biomedical self-healing hydrogel. Furthermore, an increasing number of studies on biomedical self-healing hydrogels were focused on in vivo experiments. Rather than fundamental rat/mouse subcutaneous implantation experiments, various complex and diverse animal models have been applied to evaluate the therapeutic effects of self-healing hydrogels alone or as cell/drug carriers, including mouse unilateral ureteral obstruction model (for chronic kidney disease) [54], diabetic mouse wound healing model [55], mouse unilateral hindlimb ischemia model [56], rat myocardial infarct model [57], rat cartilage defect model [58], and zebrafish injury model [46]. The latter one was the first in vivo study using self-healing hydrogel for CNS functional repair. Although many biomedical self-healing hydrogels were invented during this period, few studies were conducted using neural-related cells or relevant animal models.

Biomedical self-healing hydrogels started to emerge as desirable candidates in areas such as tissue engineering and regenerative medicine since 2015. Based on the blossoming self-healing mechanism, researchers have started to concentrate on the preparation of new materials as main chains or crosslinkers and try to introduce composite/hybrid mechanisms, which allows for the multifunctionalization of biomedical self-healing hydrogels. Hydrogels for neural tissues have also been explored to support the growth of different neural cells with appropriate strength for neurons (~ 0.1–1 kPa) and glial cells (~ 7–10 kPa) [59], as well as preferably with bioactivity to assist in tissue regeneration or functional repair [60, 61]. Additionally, due to the specificity of the brain, large incisions are an essential problem to overcome when biomedical self-healing hydrogels are used for practical clinical applications. Injectable self-healing hydrogels as potential implants have emerged as a promising strategy for treating brain disorders via minimally invasive surgery [62] and have exhibited good performances in the treatment of different CNS diseases in animal models during recent years [21, 22, 63]. It is worth mentioning that supramolecular peptide self-healing hydrogel, being the first developed category of neural-related self-healing hydrogel [27], has recently been applied in the treatment of brain/neural diseases. At the current stage of development, the innovative application of supramolecular peptide self-healing hydrogels in brain/neurological diseases was mainly achieved by synthesizing new peptide sequences or mimicking/modifying functional human peptides [64, 65]. However, no clinical application of biomedical self-healing hydrogels has been reported so far, which is bound to be the next step for researchers.

## Rational design of biomedical self-healing hydrogels

**Fig. 1:**
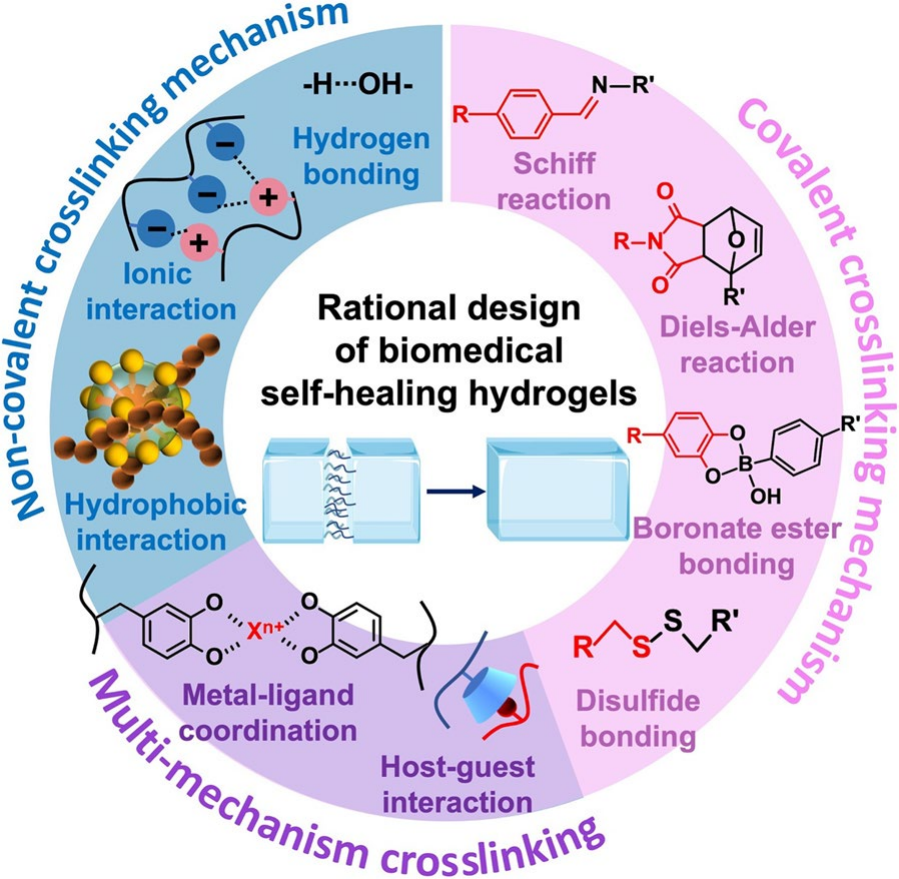
Schematics of chemistries and mechanisms for rational design of biomedical self-healing hydrogels, including covalent crosslinking mechanism, non-covalent crosslinking mechanism, and multi-mechanism crosslinking

The decisive factor in the rationalized design of biomedical self-healing hydrogels is the crosslinking mechanism, i.e., reversible chemical covalent bonding crosslinking and physical non-covalent interactions, as shown in Fig. 1. The common reversible covalent bonds include Schiff reaction, Diels–Alder reaction, boronate ester bonding, and disulfide bonding. Besides these chemical bonds, some non-covalent physical interactions can also provide self-healing properties to biomedical hydrogels, such as hydrophobic interactions, hydrogen bonding, ionic interaction, host–guest interaction, and metal–ligand coordination. Among them, networks based on host–guest interaction or metal–ligand coordination are usually based on the assembly/crosslinking of two or more chemicals through non-covalent interactions such as van der Waals forces, hydrogen bonding, electrostatic interactions, and hydrophobic interactions, i.e., multi-mechanism crosslinking.

### Non-covalent crosslinking mechanism

#### Hydrogen bonding system

Biomedical hydrogels that provide self-healing properties through hydrogen bonding are achieved by strong intermolecular interactions between the protonated polar functional groups at the end of the molecular chain of the material and the same or other polar functional groups. The self-healing ability of biomedical hydrogels depends on the degree of protonation and the chemical environment in which the polar functional groups are protonated [66]. To achieve efficient hydrogen bonding in hydrogel networks, it is necessary to reduce steric hindrance in the polymer chains and to adjust the proportion of hydrophobic and hydrophilic segments in the polymer chains to reach a balanced state. The possibility of hydrogen bond formation is presumed to be higher for gels containing abundant free hydroxyl, cyclooxy, carbonyl, and carboxyl groups versus those with chemical covalent bonds [67].

**Fig. 2:**
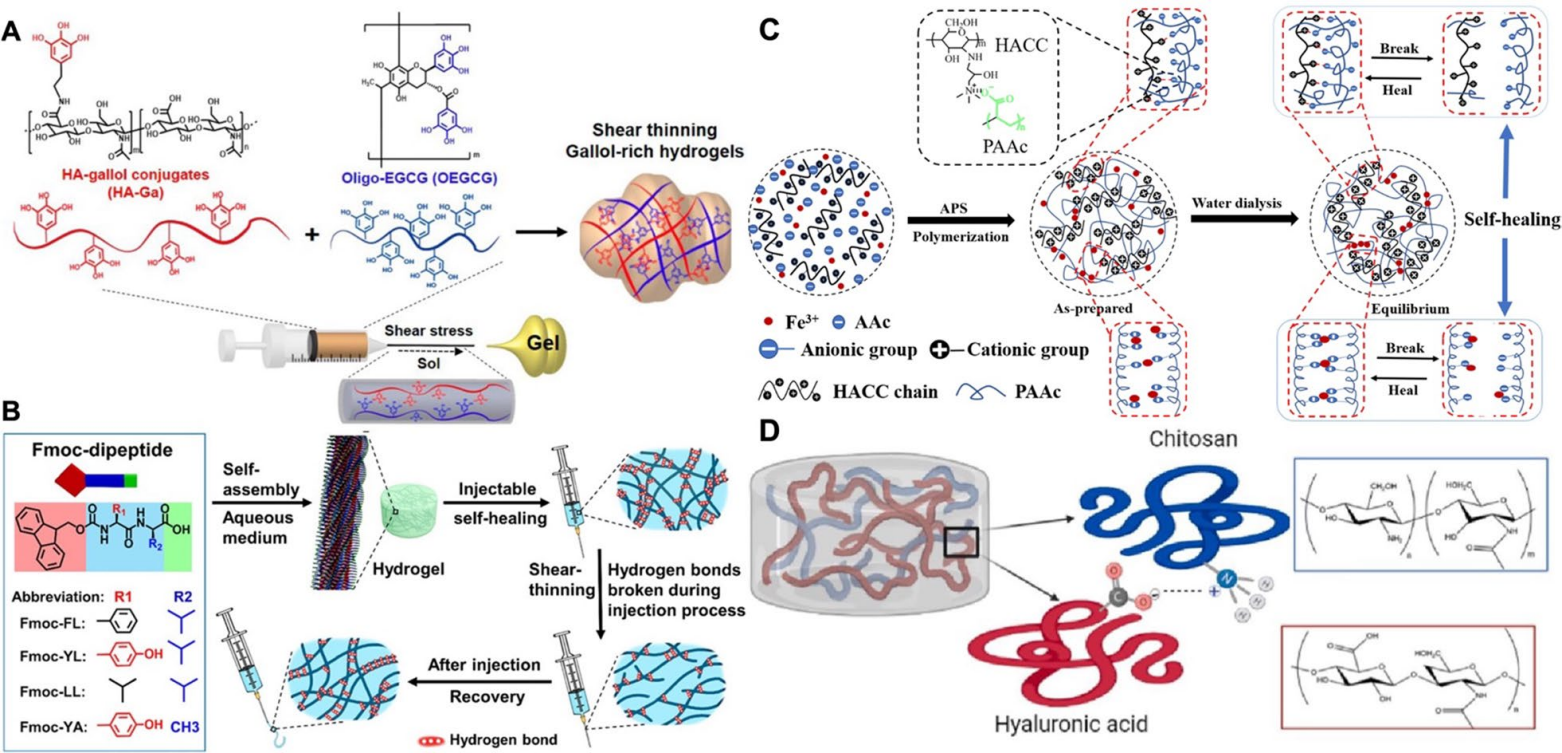
**A** Schematic illustration for preparing self-healing hydrogels of HA-gallol/gallol-rich oligomeric crosslinker (OEGCG) with shear-thinning property. Reprinted with permission from [70]. Copyright © 2017 American Chemical Society. **B** Schematic illustration of Fmoc-dipeptide self-assembly hydrogels with shear-thinning and self-healing properties. Reprinted with permission from [71]. Copyright © 2020 American Chemical Society. **C** Schematic diagram of the preparation of the dual ionic crosslinking hydrogel and a possible network structure of the hydrogel. Reprinted with permission from [72]. Copyright © 2019 Springer Science Business Media, LLC, part of Springer Nature. **D** Scheme of electrostatic interactions between hyaluronic acid and chitosan. Reprinted with permission from [75]. Copyright © 2021 Elsevier B.V

The earliest self-healing hydrogels based on polyvinyl alcohol (PVA) were prepared using a freeze–thaw method that relied on hydrogen bonding. Subsequently, strategies such as dual hydrogen bonding physical crosslinking [68] and modification of PVA [69] were developed to improve PVA crosslinking. However, due to the use of freezing conditions in the process, the biomedical applications of PVA-based self-healing hydrogels are limited. Especially, PVA-based self-healing hydrogels cannot be directly loaded with cells in situ. Additionally, these biomedical hydrogels often incorporate other chemicals to enhance the hydrogen bonding network, such as tannic acid [68], 2-ureido-4-pyrimidone (UPy) [42], and gallic acid [70]. Shin et al. [70] developed a hydrogel by synthesizing gallol-conjugated hyaluronic acid (HA) and incorporating a gallol-rich crosslinker with shear-thinning behavior and self-healing properties, which are attributed to the strong hydrogen bonding interactions between gallol-gallol and gallol-HA (Fig. 2A). In addition to the above-mentioned hydrogels, self-assembled hydrogels with self-healing ability have received much attention due to their good biocompatibility and flexible mechanical properties. For example, Ren et al. [71] proposed a hydrogen-bonding-based self-healing dipeptide hydrogel composed of 9-fluorenylmethoxycarbonyl (Fmoc) conjugated with short peptide or dipeptide with self-assembly, as shown in Fig. 2B. The shear thinning, self-healing and even mechanical properties of such hydrogel can be regulated by adjusting the design of peptide sequence to change the strength of inter-/intra-molecular interactions.

#### Ionic interaction system

Hydrogels formed by non-covalent crosslinking interactions of electrostatic forces have also been identified to own self-healing capability. When electrostatic crosslinked hydrogels are cut into two pieces, the two pieces can automatically recover themselves within a period of time. The process of cross-sectional healing of two hydrogels has been described as zwitterionic fusion that occurs in charged polymers and ions [72], polyelectrolytes [73], or polyampholytes [74]. For instance, a tough, transparent, and self-healing hydrogel was produced successfully using a bionic cross-linking strategy [72], as shown in Fig. 2C. In particular, the hydrogel is formed by in situ polymerization and crosslinking of acrylic acid in a mixture of 2-hydroxypropyltrimethylammonium chloride chitosan (HACC) and iron ions (Fe^3+^). The ionic bonding between the positively charged HACC and the oppositely charged poly(acrylic acid) (PAAc) in the biocompatible self-healing hydrogel served as the first ionic crosslinking site, and the ionic interaction between Fe^3+^ and the carboxyl groups served as the second ionic crosslinking site. The dual reversible electrostatic crosslinking network endowed the hydrogel with suitable mechanical properties and self-healing ability. Although such strategies are simple and effective, the metabolic and toxicity concerns of metal ions in biological applications cannot be ignored. Maiz-Fernández et al. [75] developed a self-healing hydrogel based on reversible polyelectrolyte complexes, composed of HA and chitosan (Fig. 2D). After mixing two polymers and precipitating for 2 h, a hydrogel was resulted with self-healing capability, shear-thinning behavior, 3D printability, adhesiveness, and cell compatibility. In addition, polyampholytes can form mechanically tunable self-healing hydrogels through electrostatic interactions between randomly dispersed cationic and anionic groups in the polymer [76]. The ionic bonding in polyampholyte hydrogels contains strong and weak bonds to maintain the shape and to enhance shock absorption and self-healing, respectively. Besides, the polyampholyte hydrogels with more hydrophobic features presented robust and poor self-healing properties, while the more hydrophilic polyampholyte hydrogels exhibited soft and good self-healing properties [74].

#### Hydrophobic interaction system

**Fig. 3:**
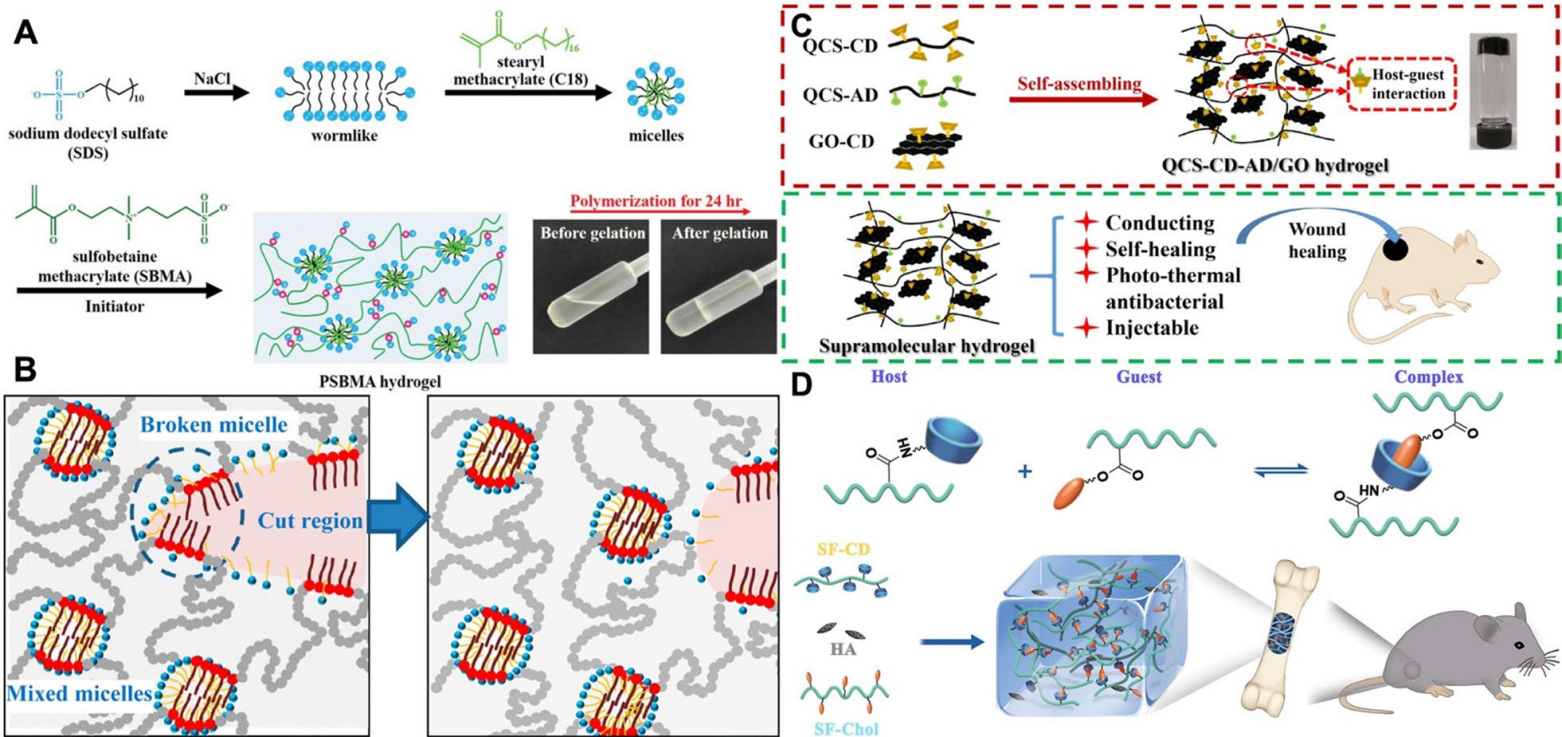
**A** Synthesis scheme for the hydrogels displaying the formation of hydrogels. Reprinted with permission from [81]. Copyright © 2022 Wiley‐VCH GmbH. **B** Cartoon showing the self-healing mechanism of micellar hydrogels indicating the gel region before and after healing. Reprinted with permission from [84]. Copyright © 2016 American Chemical Society. **C** Schematic illustration for preparation of supramolecular hydrogel via host–guest self-assembly of QCS-CD, QCS-AD, and GO-CD polymers with the application in wound healing. Reprinted with permission from [90]. Copyright © 2020 Elsevier B.V. **D** Schematic of supramolecular hydrogel formation through host–guest complexation and its application as bone graft for promoting bone regeneration. Reprinted with permission from [91]. Copyright © 2020 Elsevier B.V

Hydrophobic interactions, as a typical physical non-covalent crosslinking, are a consequence of the aggregation of hydrophobics in aqueous media that play an important role in the self-healing process of biocompatible soft materials [77]. Non-polar molecules or hydrophobic segments tend to aggregate in aqueous solutions to minimize exposure to water. Generally, the structure of these hydrophobic conjugates is thixotropic with reversible networks, and these dynamic structural properties allow biomedical self-healing hydrogels to recover efficiently in response to damage [78]. Among many reported self-healing hydrogels based on hydrophobic interactions, surfactant micelles [79] or liposomes [80] have often been used as crosslinking points to construct polymer chains containing both hydrophilic and hydrophobic monomers. Jiang et al. [81] prepared micellar polymerized self-healing biomedical hydrogels using small portions of the hydrophobic monomer searyl methacrylate (C18) and the hydrophilic monomer sulfobetaine methacrylate (SBMA) in the presence of sodium dodecyl sulfate (SDS) surfactant, as shown in Fig. 3A. The polymeric-like surfactant self-assembled into worm-like micelles in saline solution and generated interconnected spherical hybrid micelles composed of hydrophobic junctions between the copolymer chains and the hydrophobic domain of SDS, which functioned as physical crosslinks for the resulting hydrogels [82, 83]. The self-healing process of such hydrogels, as illustrated in Fig. 3B, benefits from the intra- and interlayer fluidity of the hybrid micelles that drives the injured area to remodel into a circular hole before healing gradually and completely [84]. In addition, several studies have discussed the differences in self-healing behavior and mechanical properties of hydrogels with or without the use of surfactants [85, 86]. The results suggest that SDS plays an important role in these hydrogels, by affecting their mechanical properties and self-healing characteristics. While self-healing hydrogels based on hydrophobic interactions have been widely reported for biomedical applications, their use requires careful consideration of biodegradability, byproducts, biotoxicity, and in vivo metabolism due to the utilization of synthetic polymers and possibly in situ polymerization reactions.

### Multi-mechanism crosslinking

#### Host–guest interaction system

Host–guest interaction, a type of supramolecular interaction, is a force generated when two or more chemicals are assembled through non-covalent interactions (e.g., hydrogen bonding, electrostatic gravity, hydrophobic interactions, etc.) [87]. In host–guest chemistry, the main part of the macrocycle is inserted inside the guest part to form a unique inclusion structure. In host–guest interaction-mediated biomedical self-healing hydrogels, the vast majority of studies have focused on the use of macrocyclic cyclodextrins (CDs) and, to a lesser extent, cucurbiturils (CBs) as the host part because of their good biocompatibility and ease of functionalization [88]. However, most of the reported host-object action-based biomedical self-healing hydrogels have been used for drug release, 3D bioprinting, bone tissue engineering, and wound healing [89], and lack the applications in neural-related fields, which may be attributed to the fact that the modulus of all these hydrogels are relatively high and exceeds the preferable modulus of neural/brain tissue. For instance, Guo and colleagues prepared a series of self-healing, antibacterial, and electroconductive hydrogels for wound healing by mixing the aqueous solutions of quaternized chitosan-graft-cyclodextrin (QCS-CD), quaternized chitosan-graft-adamantane (QCS-AD), and graphene oxide-graft-cyclodextrin (GO-CD) via host-host compounding (Fig. 3C) [90]. Bai et al. [91] reported self-healing hydrogels based on filamentin protein (SF) grafted β-CD (SF-CD, host molecule) and SF grafted cholesterol (SF-Chol, guest molecule) composited with bioactive inorganic hydroxyapatite nanoparticles as bone regeneration scaffolds. Such composite hydrogels, as shown in Fig. 3D, are proved to be highly effective in promoting cell proliferation and osteogenic differentiation in vitro as well as accelerating bone regeneration in vivo.

#### Metal–ligand coordination system

**Fig. 4:**
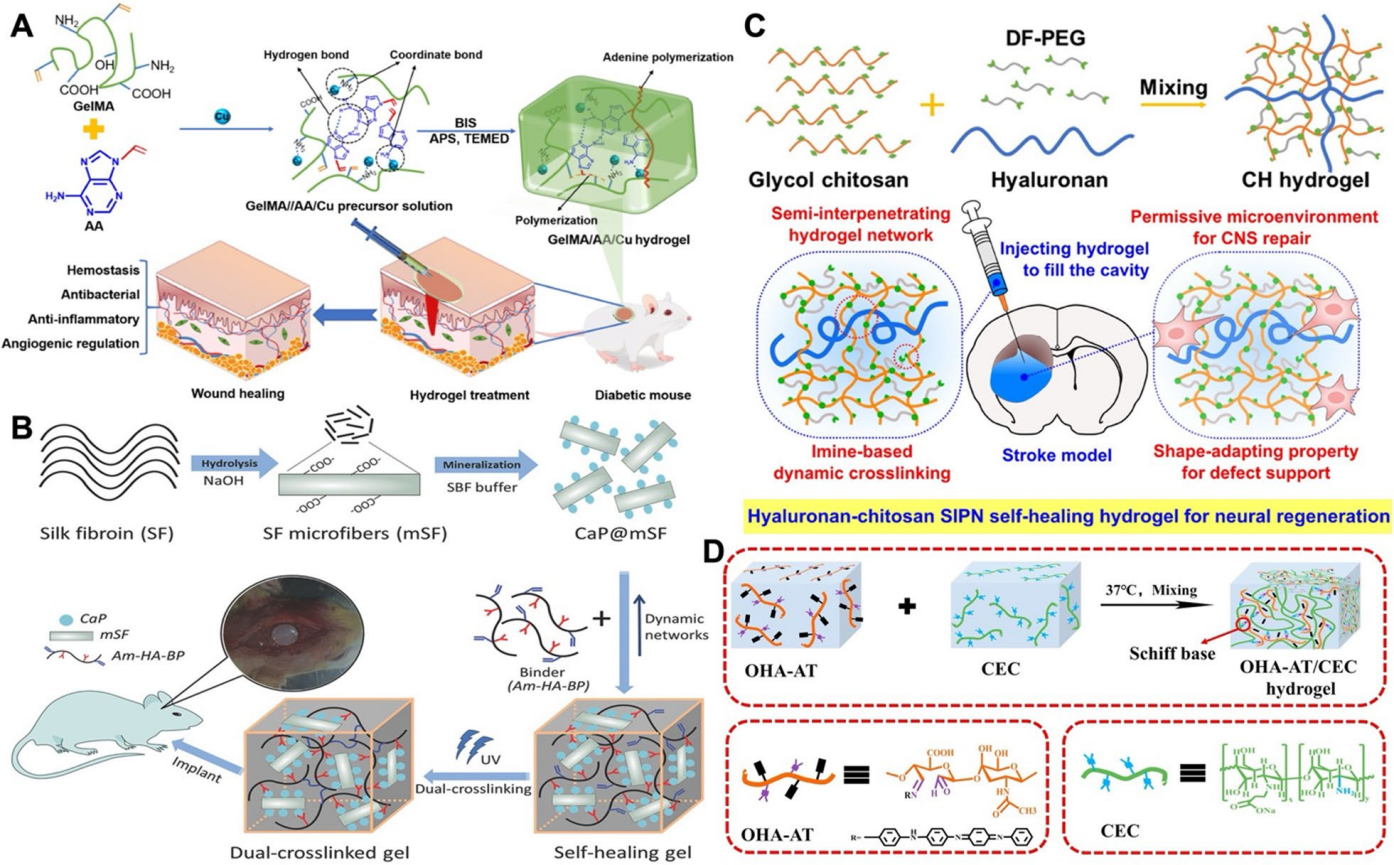
**A** Preparation of GelMA/AA/Cu^2+^ hydrogels and biomedical applications in a mouse wound healing model. Reprinted with permission from [94]. Copyright © 2022 Elsevier B.V. **B** Schematic of the fabrication of self-healing SF-based hydrogel for bone regeneration. SBF: simulated body fluid. Am-HA-BP: BP modified acrylamide-grafted hyaluronan biopolymer. Reprinted with permission from [95]. Copyright © 2017 Wiley‐VCH GmbH. **C** Schematic of the formation of CH hydrogel and the potential biomedical application. Reprinted with permission from [21]. Copyright © 2020 American Chemical Society. **D** Synthesis scheme of OHA-AT/CEC hydrogel. Reprinted with permission from [99]. Copyright © 2019 Elsevier B.V

Metal–ligand coordination chemistry has been used since its discovery to explain biological phenomena in nature [92]. Most notably, the self-repairing properties of mussel adhesion proteins are triggered by the alkaline pH condition in seawater and oxidant cations, leading to the formation of metal complexes between unoxidized catechols and the polyvalent cations in seawater [93]. Self-healing hydrogels in this category, which are dominated by a metal–ligand mechanism and usually accompanied by hydrogen bonding, have good adhesion properties. Therefore, these self-healing hydrogels are mainly used for wound healing applications. Very recently, a series of self-healing, adhesive, and antimicrobial hydrogels based on gelatin methacrylate (GelMA), adenine acrylate (AA), and copper ions (Cu^2+^) were prepared as shown in Fig. 4A and their ability to promote diabetic wound healing was confirmed by in vivo tests [94]. Such hydrogel composed of multiple crosslinking mechanisms presents sufficient mechanical strength. Therefore, biomedical self-healing hydrogels containing metal–ligand bonding are more suitable for bone tissue engineering and are rarely used in neurological applications. Shi et al. [95] provided a general strategy for the in situ assembly of self-healing SF-based hydrogels under physiological conditions based on dynamic metal-bisphosphonate (BP) coordination between SF microfibrils (mSF) and polysaccharide binders without any chemical and physical stimulation. Calcium phosphate (CaP) particles coated with bio-inspired mineralization on mSF (CaP@mSF) were the source of metal ions in the hydrogel system, as demonstrated in Fig. 4B. The double crosslinked SF-based hydrogel supported in vitro stem cell proliferation and accelerated bone regeneration in rats with critical cranial bone size defects without additional morphogenetic factors. Although current research results on many hydrogels based on metal–ligand coordination chemistry show promising results through in vitro and in vivo studies, the potential toxicity of metal ions, the dose used, and the alkaline environment of some hydrogels remain challenges and need to be further addressed.

### Covalent crosslinking mechanism

#### Schiff reaction system

Schiff base linkages are a category of dynamic covalent bonds formed by the reaction of amino groups with carbonyl groups. Schiff base linkages endow hydrogels with self-healing ability by uncoupling and recoupling of reversible bonds in the hydrogel network under mild conditions [96]. Because Schiff reaction mimics the healing mechanism existing in living organisms, Schiff base self-healing hydrogels have considerable applications in various biomedical fields [20, 97]. Liu et al. [21] prepared a self-healing hydrogel (CH hydrogel) with a semi-interpenetrating polymer network (SIPN) based on Schiff reaction crosslinking, composed of GC, DFPEG, and HA (Fig. 4C). Such hydrogel was confirmed to have good injectability, compact nanostructure, and porous microstructure. Meanwhile, the SIPN hydrogel formed by HA and Schiff base bonded network provided an adaptive environment for cell expansion and migration as well as potentially served as an injectable defective support for the repair of the CNS. In addition to semi-interpenetration or compounding that are commonly used strategies to impart multiple capabilities to hydrogels based on Schiff linkage networks, functionalized main chains or crosslinkers are also used to provide multifunctionality to hydrogels [11, 98]. Qu et al. [99] developed a pH-responsive self-healing injectable hydrogel based on *N*-carboxyethyl chitosan (CEC) and oxidized hyaluronic acid-grafted aniline tetramer (OHA-AT), as demonstrated in Fig. 4D. HA was oxidized and modified with conductive oligomers to offer conductivity (~ 0.42 mS/cm) to the hydrogel while acting as a crosslinker. The hydrogel has also been proved to be a practical drug carrier to repair full-thickness skin defect. Design of biomedical self-healing hydrogels based on Schiff base bonds inevitably requires consideration of mechanical strength, and most of the published self-healing hydrogels have modulus values falling into the range of about 10 kPa, which can limit the scope of their targeted applications [62].

#### Diels–Alder reaction system

**Fig. 5:**
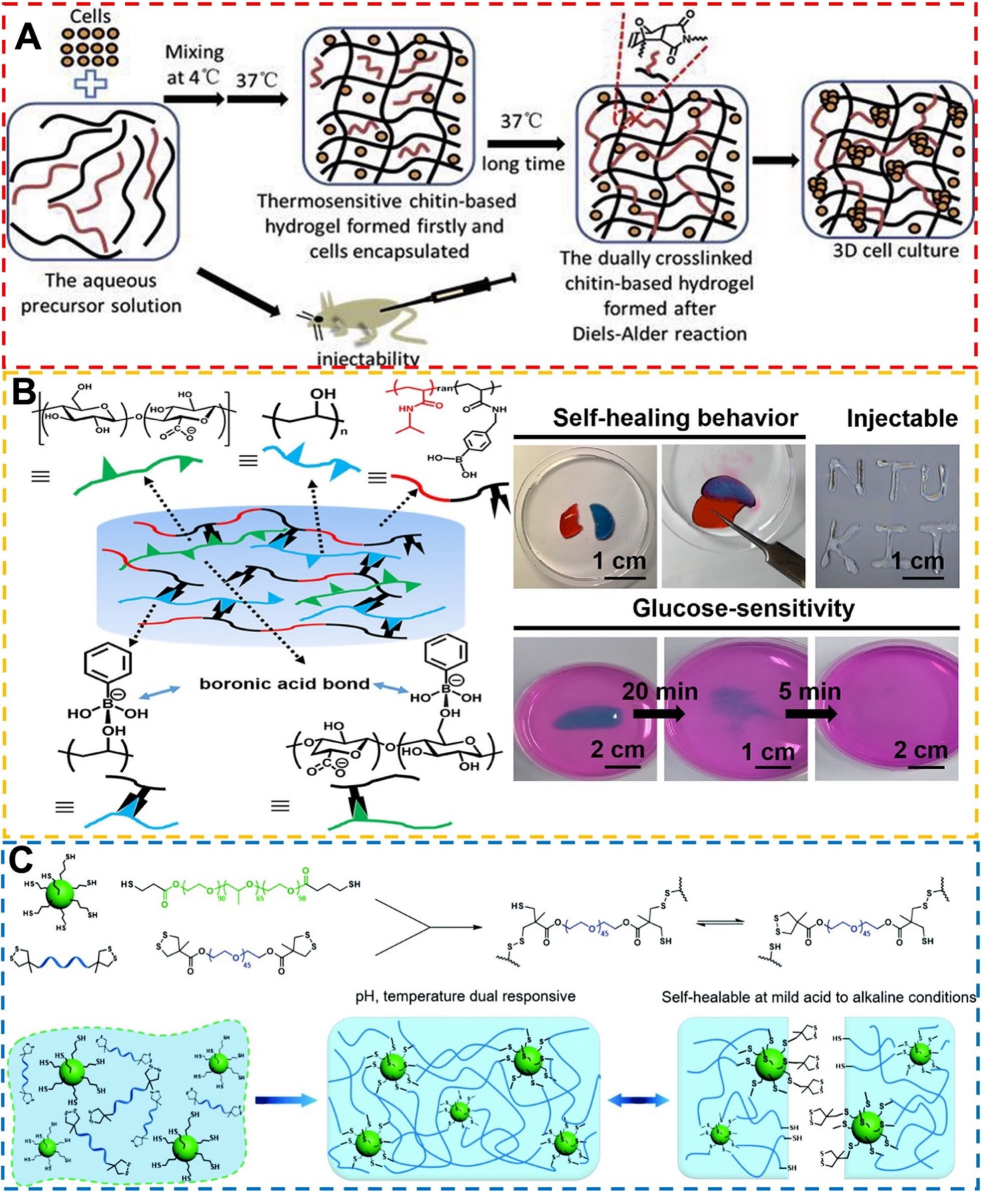
**A** Preparation of the double crosslinked self-healing hydrogels with injectability. Reprinted with permission from [102]. Copyright © 2019 Elsevier B.V. **B** Preparation of boronic ester-based self-healing hydrogel and macroscopic observation for the self-healing and glucose-sensitive behaviors of hydrogels. Reprinted with permission from [106]. Copyright © 2020 Elsevier Ltd. **C** Schematic illustration of the design, synthesis and application of the injectable self-healing hydrogel using reversible thiol/disulfide exchange reaction. Reprinted with permission from [109]. Copyright © 2017 Royal Society of Chemistry

Another important dynamic covalent chemistry in biomedical self-healing hydrogels is the thermally reversible Diels–Alder (DA) reaction [100]. However, the biomedical application of DA reaction is limited because DA bonds require high temperature and long time for cleavage and re-formation of self-healing properties [101]. Therefore, DA-based hydrogels need to be combined with other crosslinking networks, such as hydrogen bonding network, to prepare dual-crosslinked self-healing hydrogels to alleviate the temperature dependence of the DA reaction. As an example, an injectable double crosslinked hydrogel combining the thermal gelation of temperature-sensitive furyl-modified hydroxypropyl chitin and DA reaction via maleimide-terminated PEG crosslinker was prepared under physiological conditions (Fig. 5A) [102]. Nevertheless, the biomedical application of this hydrogel mainly benefits from the rapid crosslinking of the physical crosslinking network to achieve in situ gelation of the hydrogel precursor after injection, while introducing the DA reaction to slowly complement the physical crosslinking network for its effective retention at the injection site. Self-healing hydrogels prepared using a similar dual crosslinking strategy are mainly used as drug/cell carriers, but the release mechanism is basically achieved through degradation [103]. DA-based self-healing hydrogels are not competitive enough for applications in the biomedical field compared to the crosslinking mechanisms that can be performed under mild conditions.

#### Boronate ester bonding system

Some diols and boronic acids can be complexed to form reversible boronic esters in aqueous solutions [104]. The stability of the borate compounds depends strongly on the pH value. Only when the pH is higher than the pKa of the diol (usually at alkaline pH) can the ionization of the diol be triggered, resulting in the formation of a stable boronic ester complex. However, the unreacted boronic acid monomers are notably biotoxic, leading to concerns about the use of these hydrogels in biomedical applications [105]. Besides, the glucose-sensitive characteristics of boronic ester-based hydrogels makes these hydrogels a candidate to be developed as a sacrificial material for 3D bioprinting. Tsai et al. [106] developed a self-healing injectable hydrogel based on boronic acid esters with tunable mechanical properties, as shown in Fig. 5B. The hydrogel combines poly(*N*-isopropylacrylamide) and borate functional groups through pentafluorophenyl ester functionalization with the addition of cellulose nanofibers (CNFs) to modulate the rheology. This printable borate ester hydrogel can be expected to support the bioprinting of vascular tissue engineering multichannel structures. Nevertheless, the preparation and application of borate ester-based self-healing gels still suffers from many limitations, especially the difficulty of controlling alkaline or acidic reaction conditions and the incompatibility with biologically neutral environments [62]. Thus, further studies on the formation and application of boronic ester-based chiral gels under physiological conditions are warranted.

#### Disulfide bonding system

Disulfide-thiol exchange reactions can explain the dynamic reversible covalent chemistry of disulfide bonds, so they greatly depend on pH values and redox potentials [107]. Disulfide bonds undergo exchange reactions in which S–S neighboring bonds are damaged and reorganized through ionic intermediates [108]. Disulfide bonding networks usually also combine other crosslinking methods, such as hydrogen bonding, metal–ligand coordination chemistry, and Schiff reaction, to compensate for the temperature and pH sensitivity [18]. An injectable thermoresponsive hydrogel that can self-heal under weakly acidic to alkaline conditions was prepared by simply mixing thiol-functionalized F127 with a dithiolane-modified PEG [109]. The disulfide bond exchange reaction and F127 hydrogen bonding crosslinking of this hydrogel network were illustrated schematically in Fig. 5C. The formation of disulfide bonds in hydrogels ensures biocompatibility without degradation. However, this type of hydrogel introduces disulfides in the network to complete the crosslinking, and the toxicity of small molecule upon degradation of the self-healing hydrogel is an important issue to be considered. Probably for the above reasons, disulfide bond-based self-healing hydrogels are typically used in drug delivery and antibacterial applications in the biomedical field at present [11].

## Current status of self-healing hydrogel for the brain disease therapy

**Fig. 6:**
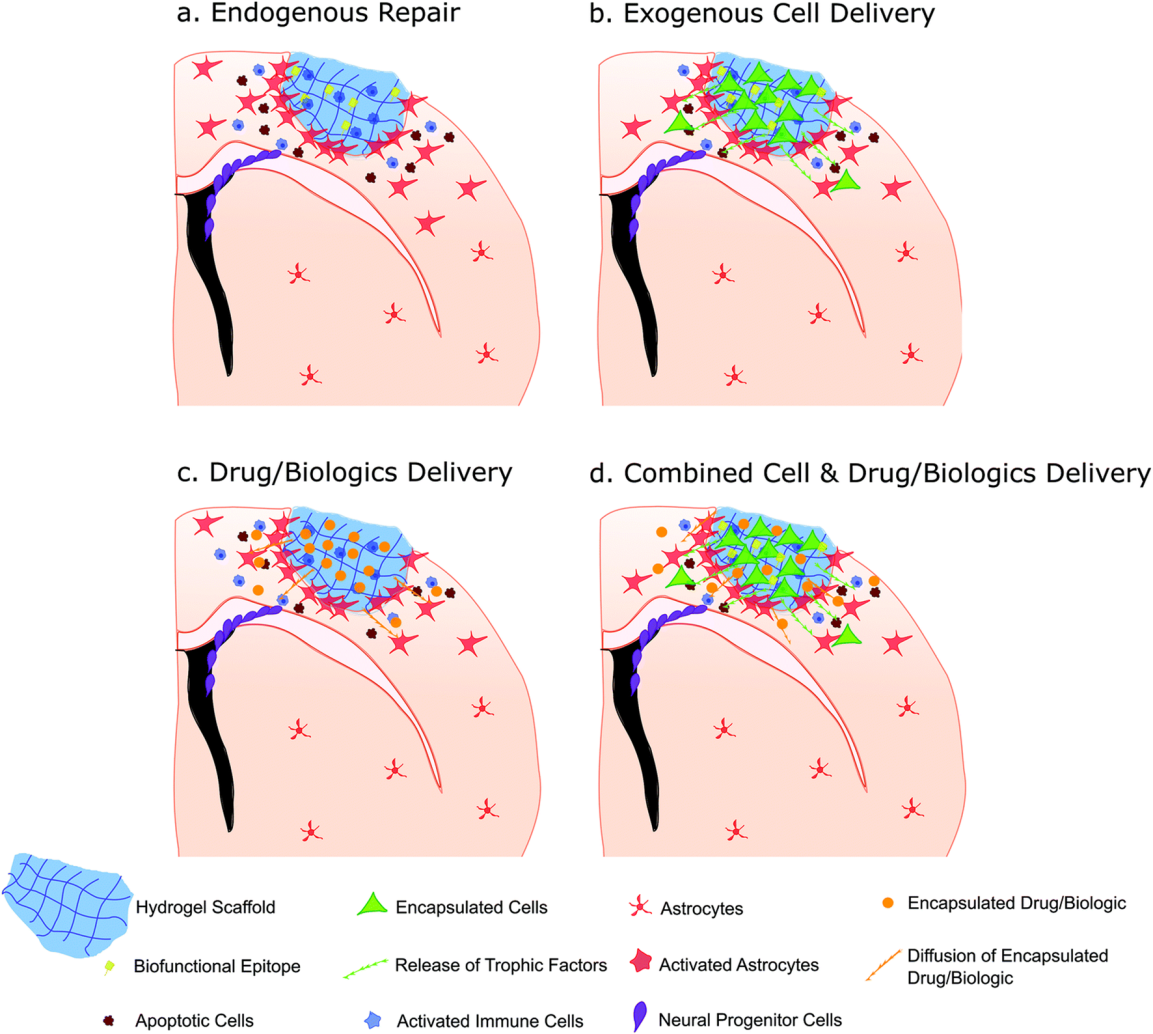
Schematic of the potential in vivo applications of injectable hydrogels in the treatment of stroke. Reprinted with permission from [110]. Copyright © 2019 Royal Society of Chemistry

There are currently four strategies to utilize self-healing hydrogel for brain disease treatments, including endogenous repair, exogenous cell delivery, drug/biologics delivery, as well as combined cell and drug/ biologics delivery, as shown in Fig. 6 (taking the stroke as an example [110]). The endogenous repair strategy proposes that the injection of functional self-healing hydrogel into the target site can induce endogenous repair mechanisms, such as angiogenesis and nerve regeneration. The exogenous cell delivery strategy refers to the encapsulation of cells with injectable self-healing hydrogel to provide a proper 3D environment after injection into the brain where the encapsulated exogenous cells may release therapeutic nutrients into the surrounding environment to aid regeneration. The drug/biologics delivery strategy indicates that the injectable self-healing hydrogel is employed as a carrier for relevant CNS diseases to facilitate the controlled release of drug/biologics within a specific location. The delivery strategy of combining exogenous cells and drug/biologic agents is to leverage the effects of both encapsulants in the self-healing hydrogel in anticipation of dual or mutually reinforcing effects. The current status is summarized in Table 1 and described below in detail according to various brain injury and diseases.

**Table 1:** The representative self-healing hydrogels utilized for treating various brain diseases

Type of brain diseases	Hydrogel composition	Self-healing mechanism	Study design	Role of hydrogel	Refs.
Ischemic stroke	GC/DFPEG	Schiff base	Rat MCAO model	Cell carrier (rat BMSC)	[118]
Hemorrhagic stroke	GC/DFPEG/HA	Schiff base with SIPN	Rat ICH model	Tissue scaffold	[21]
Parkinson’s disease	l-glutamine amide derivative/benzaldehyde	Supramolecular interaction and Schiff base	Mouse PD model	Drug carrier for nasal delivery	[131]
Parkinson’s disease	CMC/OTA@Au	Schiff base	Rat PD model	Tissue scaffold	[132]
Alzheimer’s disease	No practical self-healing hydrogel has obviously treatment efficiency verified through in vivo test for AD				
Traumatic brain injury	HA-PBA/Gel-Dopa	Boronate ester	Rat brain TBI model	Tissue scaffold	[160]

### Stroke

#### Ischemic stroke

Stroke is the leading cause of death and disability worldwide, with over 62% of all strokes being ischemic for global incidence [111]. The feature is the occlusion of blood vessels, particularly the middle cerebral artery from the internal carotid artery, resulting in inadequate perfusion of oxygenated blood and inadequate nutritional supply to the brain [112]. Ischemic/hypoxic condition triggers a series of neuropathological pathways in hypoperfused tissues that leads to substantial brain loss and infarction, further resulting in impairment of brain function and severe neurological and motor dysfunction [113]. The current therapeutic approach to ischemic stroke aimed at symptomatic relief is incapable of achieving neuronal regeneration and neurological recovery, and therefore new therapeutic strategies are urgently needed [114, 115]. While research has been vigorously promoted, the complexities of brain physiology and the harsh environment of the ischemic penumbra pose potential challenges to successful clinical translation [116]. Expressly, the efficacy of stem cell therapy for ischemic stroke is limited by insufficient cell migration to the infarct region, high mortality of transplanted exogenous cells, and poor integration of transplanted exogenous cells into the damaged brain tissue [117].

**Fig. 7:**
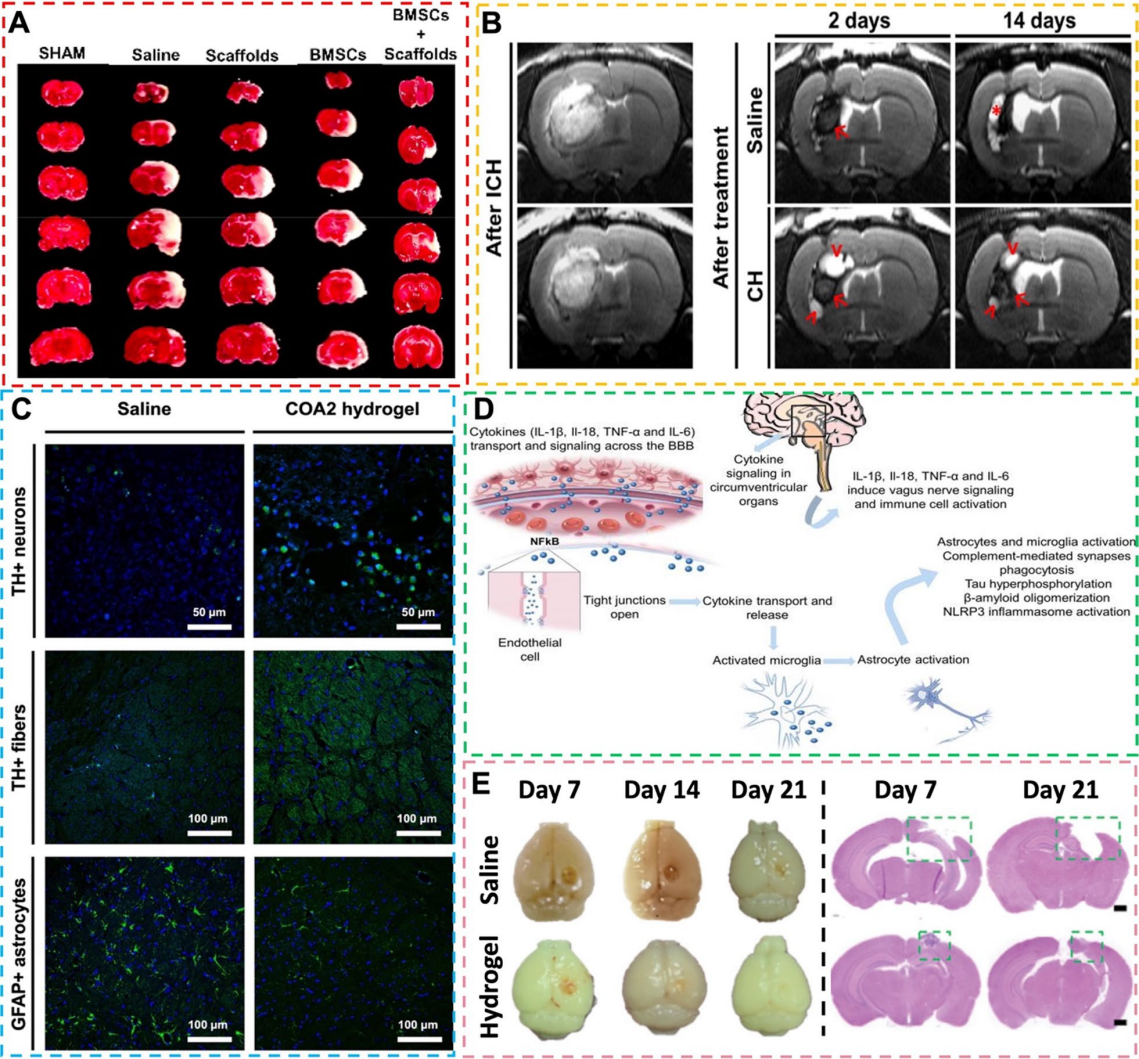
**A** BMSCs loaded in composite self-healing hydrogel (“scaffolds” in figure) ameliorated ischemic stroke. The effects of BMSCs and BMSC-loaded self-healing hydrogel (“BMSCs + scaffolds” in figure) were assessed by TTC staining. Reprinted with permission from [118]. Copyright © 2023 Pei et al. First published by Elsevier Ltd. **B** Efficacy of the CH hydrogel in alleviating the brain atrophy and neurological deficits in the ICH rat model. Reprinted with permission from [21]. Copyright © 2020 American Chemical Society. **C** The capabilities of conductive self-healing hydrogels (COA2 hydrogel) injected in the brain of PD rats to protect dopaminergic neurons/fibers, to reduce neural inflammation, and to improve motor functions. TH: tyrosine hydroxylase. GFAP: glial fibrillary acidic protein. Reprinted with permission from [132]. Copyright © 2023 Xu et al. First published by BioMed Central Ltd. **D** Schematic representation of the systemic process of inflammation in AD neuroinflammation. Reprinted with permission from [139]. Copyright © 2021 Cunha et al. First published by Dove Medical Press Ltd. **E** Tissue regeneration status of TBI rat brain after the self-healing hydrogel injection for 21 days. Reprinted with permission from [160]. Copyright © 2022 Wiley‐VCH GmbH

Self-healing hydrogel as a promising implant material has been shown to improve ischemic stroke when serving as a stem cell carrier. Pei et al. [118] investigated the efficacy of bone mesenchymal stem cells (BMSCs)-loaded GC-DFPEG self-healing hydrogel composited with nanofibers through a rat middle cerebral artery occlusion (MCAO) model for the treatment of ischemic stroke. After implantation for 14 days, a substantial decrease in the area of cerebral ischemia among rats receiving the BMSC-loaded self-healing hydrogel was visualized under the 2,3,5-Triphenyltetrazolium chloride (TTC) staining (Fig. 7A). Behavioral evaluation and histological analysis also supported that rats receiving cell-containing self-healing hydrogel exhibited significant symptom relief in various behavioral patterns, lower brain inflammation, and more neuroregeneration and angiogenesis in the ischemic areas of the brain. Other studies have confirmed that using NSC-containing Schiff base crosslinked self-healing hydrogel implanted into the MCAO rat brain can induce long-distance migration of neuroblasts into the olfactory bulb, where they differentiate into local neurons [119, 120]. Based on the current literature, the majority of self-healing hydrogels serve as stem cell carriers in treating the ischemic stroke. The design of self-healing hydrogels as implants in ischemic stroke disease requires to consider and focus on their expected effects on promoting angiogenesis, neuronal regeneration, neuroprotection, and neural cell migration in the area of cerebral ischemia.

#### Hemorrhagic stroke

ICH is the second most common subtype of stroke after ischemic stroke, with an average mortality rate of approximately 40% within 1 month of illness [121, 122]. The current strategy for treating ICH is primarily surgical with conservative clinical management, theoretically to improve the prognosis of ICH by reducing hematoma, minimizing neurological damage, and removing harmful chemicals. However, the outcome of ICH surgery is always unsatisfactory due to postoperative rebleeding [122, 123]. Literature also confirms that early surgery does not reduce mortality or disability rates within 6 months [122]. Several factors may contribute to poor outcomes after ICH surgery, including postoperative rebleeding, low clearance rates, and surgically induced brain damage [124]. Although techniques for hematoma decompression surgery are being developed, the treatment of postoperative rebleeding remains limited [125].

Brain tissue after ICH shows neuroglial scars, which are important structures to protect the neural network from further damage but pose a major obstacle to distant axonal regeneration, as well as the formation of the lesion cavity [21]. For reducing neural scar formation and promoting neural tissue regeneration in the damaged area, the feasibility of using biodegradable support matrices, such as hydrogels, to fill the lesion cavity and to provide a biocompatible microenvironment has been supported by animal studies. A GC-DFPEG-HA hydrogel, i.e., CH hydrogel, was used as an injectable implant for filling the lesion cavity of ICH rat brain [21]. The CH hydrogel induced a moderate inflammatory response (edema, leukocyte infiltration, microglial cell proliferation, and astrocyte proliferation) and neuroglial scar formation after implantation into the rat brain. Injection of the CH hydrogel enhanced the brain cavity repair and neurobehavioral recovery after 14 days, as shown in Fig. 7B. Immunostaining analysis confirmed that CH hydrogel improved the recruitment of native NSCs and functionalization for neurogenesis in the brain. In contrast to general hydrogels, self-healing hydrogels can be injected locally after stable gelation or gelated in situ after injection to adapt to the area of tissue defect and filling. Literature on using self-healing hydrogels to treat ICH is still limited. The design of self-healing hydrogels for treating ICH necessitates consideration of biocompatibility, biodegradability, appropriate mechanical strength, hemostatic property, and the ability to promote nerve tissue repair [23, 126]. Subsequent development of self-healing hydrogels with their own therapeutic properties can be combined with cell/drug delivery to maximize the efficacy in treating ICH.

### Neurodegenerative diseases

#### Parkinson’s disease

PD is a neurological disorder that damages the dopamine-producing neurons in the brain, which affects millions of people worldwide. This disease is chronic and progressive, with symptoms including tremors, stiffness, and difficulty with movement and coordination [127]. Currently, there is no cure for PD, and the available treatments are limited in their ability to manage its symptoms. The most common treatment is medication, which can help increase dopamine levels in the brain and alleviate symptoms [128]. However, medication may lose its effectiveness over time, and it can have side effects such as nausea, dizziness, and hallucinations. Other treatments for PD include deep brain stimulation (DBS) and physical therapy. DBS involves the implantation of electrodes in the brain that deliver electrical stimulation to specific areas, helping to regulate movement and reduce symptoms [129]. However, DBS is a relatively expensive treatment and takes several months after the procedure before the full benefits of DBS are realized. The cost is a significant barrier for many patients and make them not be willing to undergo such a lengthy process. Physical therapy can help improve mobility, flexibility, and balance, but it may not be effective for patients [130]. Therefore, there is an urgent need for potential therapeutic approaches that are available to manage symptoms of PD. Recent studies have shown promising results for the use of self-healing hydrogels in the treatment of PD [22, 131, 132]. The unique property makes it a durable and long-lasting option to deliver drugs to the brain and alleviate the symptoms of PD. In one research, Wang et al. [131] have developed a self-healing hydrogel as a vehicle for nasal delivery of a neurologically-active drug levodopa. The drug successfully released from the gel into the blood and directly to the brain, which has high potential to treat PD. In other studies, conductive self-healing hydrogels incorporating bioactive molecules were verified to possess the ability of releasing both drugs and electrical signals to the brain [22, 132]. The advantages of using self-healing hydrogels with conductivities in the range of ~ 0.8 mS/cm to 13 mS/cm are their ability to deliver electrical signals to neural cells and to promote the communication of cells within the impaired brain region as well as the integration with brain tissue [133]. These hydrogels as injectable implants were evaluated to have the capabilities of improving motor function, protecting dopaminergic neurons/fibers, and reducing neural inflammation in the brain of PD rats (Fig. 7C). Overall, the design of future self-healing hydrogels with potential therapeutic effects on PD should take several important properties into consideration, especially conductivity, anti-oxidation, and anti-inflammatory.

#### Alzheimer’s disease

Alzheimer's disease (AD) is one of the world's most prominent neurodegenerative diseases and a pressing public health problem, as AD causes severe dementia for which there is no cure [134]. The initial characteristic of AD is short-term memory loss, progressing to more severe deficits due to a vicious cycle of neuronal damage [135]. Although the pathogenesis of AD is not fully known, the accumulation of misfolded Tau proteins that lead to intra-neuronal neurogenic fiber tangles, extracellular senile plaques, neuronal loss, and microglia activation is thought to be the main hallmarks of the disease [136, 137]. Further insights reveal that multiple factors associated with genetic alterations, innate immune responses, systemic neuronal inflammation, aging, and unbalanced diet may contribute to widespread neuronal degeneration, synapse loss, and diffuse brain atrophy [137, 138]. Accordingly, modulation/suppression of neuroinflammation has become a critical factor in the treatment of AD, as demonstrated in Fig. 7D [139]. The only current treatments for AD are long-term pharmacotherapy with acetylcholinesterase (AChE) inhibitors or a dual combination of AChE inhibitors and *N*-methyl-d-aspartate (NMDA) receptor antagonists, and non-pharmacological treatments with the consumption of vegetables and fruits with active substances. Drug therapy, administered orally or transdermally, requires passing through the body cycle and crossing the blood–brain barrier (BBB) to reach the CNS, with minimal actual utilization. Non-pharmacological treatments can only delay the progression of the disease as much as possible, and their effectiveness is lower than that of drugs [140, 141]. Since AD treatment requires continuous drug delivery at a specific point in the brain, the use of hydrogel material for nasal-to-brain drug delivery is a promising treatment strategy [139]. However, to date, no studies have actually used self-healing hydrogels in animal models of AD. The only studies mentioning the preparation of self-healing hydrogels with therapeutic potential for AD have only achieved the controlled release of the relevant drugs subcutaneously in animals or adhesion to mucosa, without any tests regarding the effectiveness on AD disorders [142–144]. In addition to using self-healing hydrogels as drug carriers, the introduction of bioactive materials, such as phenolic compounds and natural plant extracts, from the material design to provide self-healing hydrogel with anti-inflammatory and antioxidant capabilities can also be expected to have promising effects on AD [145]. The cationic dendrimers have also been shown to inhibit the formation of long-chain amyloid-like protofibrils, such as prions and α-synuclein, providing a choice of materials for the future design of self-healing hydrogels to treat AD [146, 147].

### Traumatic brain injury

Traumatic brain injury (TBI) is a global structural brain injury with high morbidity and mortality, resulting in hemiplegia, aphasia, coma, and even death, posing a severe threat to the quality of life of patients [148]. The pathological process of TBI includes primary and secondary damages. Primary brain injury typically represents direct damage to neural tissue from external forces such as skull deformation, brain tissue contusion, and axonal injury [149]. Secondary brain injury occurs minutes later and lasts for a long time after the injury, including ionic homeostasis, excitotoxicity, oxidative stress, inflammation, and cell death [150]. The initial damage to brain structure and function is more critical in secondary brain injury than in primary brain injury, making the prevention of the occurrence and severity of secondary injury reasonable and crucial [151]. Oxidative stress and inflammation are two of the most important mechanisms of secondary injury and are therapeutic targets for neuroprotection and neuroplasticity [152]. Although the number of studies related to TBI has increased over the past few decades, the outcomes of patients with TBI have improved significantly with surgery or medications. However, brain surgery is often complex and has many unpredictable complications, like epilepsy, hydrocephalus, coma, and possible secondary surgery [153]. On the other hand, most ROS scavengers and anti-inflammatory drugs administered orally or intravenously have so far been ineffective, mainly due to their limited diffusion across the BBB to the injury area [154]. Therefore, the exploration and development of alternative therapeutic strategies are highly desirable. With the accelerated development of tissue engineering and regenerative medicine, hydrogel has proven to own a lot of advantages as an efficient stem cell/drug delivery vehicle. Hydrogels can be designed to mimic the ECM in the brain with good biocompatibility and have also shown to be usable as a drug delivery system for neurological applications. Among these, self-healing hydrogel, with the adaptive properties and injectability, can fill the injury cavity via minimally invasive surgery to facilitate cell infiltration, neuroglial recruitment, and activation of neuroglial scars [155, 156]. The use of biomedical self-healing hydrogels as stem cell/drug carriers for TBI was the earliest strategy to be utilized, with favorable results [157]. However, very recently, researchers have noticed that using bioactive molecules to prepare self-healing hydrogels may achieve competitive therapeutic results without introducing exogenous stem cells/drugs, such as conductive hydrogels and anti-inflammatory hydrogels [63, 158, 159]. Hu et al. [160] developed a self-healing hydrogel based on dynamic borate ester crosslinking between phenylboronic acid grafted hyaluronic acid (HA-PBA) and dopamine-grafted gelatin (Gel-Dopa) for brain tissue repair by injection into the damaged area of the brain. Histological analyses revealed that the damaged part of brain tissue was significantly repaired after 21 days, whereas the adhesive, hemostatic, and antioxidant properties of the hydrogel were beneficial to the tissue repair (Fig. 7E). The bioactive self-healing hydrogel was also found to possess the ability to promote neural tissue regeneration and, more importantly, to improve motor function recovery [63, 161]. Future self-healing hydrogels for treating TBI that are expected to be translated into clinical trials should be designed to meet the mechanical properties of brain tissues while also having multiple functional purposes, specified conductivity, anti-inflammatory properties, antioxidant properties, tissue adhesion, and hemostatic properties.

## Conclusion and perspective

Self-healing hydrogel has shown great potential in regenerative medicine through a range of approaches such as minimally invasive surgery. In recent years, the research on the application of self-healing hydrogels in brain/neural-related fields has become increasingly popular. However, there has not been a systematic overview of the mechanisms of interaction and design criteria for self-healing hydrogels that can be applied to different brain diseases. This review summarizes the development of self-healing hydrogels for biomedical applications and the design strategies according to various crosslinking (gel formation) mechanisms. Current advances in using self-healing hydrogels to treat brain diseases and the underlying design principles are also outlined. Meanwhile, many new issues may emerge if this new therapeutic approach is to be translated into humans. In human brain, the distance may be too far for endogenous cells to migrate effectively and the amount of implanted hydrogel may be too minimal to be effective. Despite at an early stage of development, the potential of self-healing hydrogel in treating brain diseases has attracted much recent interests. Presumably, when self-healing hydrogels are implanted in humans, cells or drugs/active substances should follow. This new therapeutic strategy is well worthy of being translated into the human body clinically in the future and may herald a new era in the treatment of brain diseases plaguing people for centuries without eradication.

## Data Availability

Not applicable.

## Abbreviations

ECM: Extracellular matrix
CNS: Central nervous system
ICH: Intracerebral hemorrhage
PD: Parkinson's disease
3D: Three-dimensional
NSC: Neural stem cell
DFPEG: Difunctionalized polyethylene glycol
GC: Glycol chitosan
DnL: Dock-and-Lock
PVA: Polyvinyl alcohol
UPy: 2-Ureido-4-pyrimidone
HA: Hyaluronic acid
Fmoc: 9-Fluorenylmethoxycarbonyl
HACC: 2-Hydroxypropyltrimethylammonium chloride chitosan
Fe^3+^: Iron ion
PAAc: Poly(acrylic acid)
C18: Searyl methacrylate
SBMA: Sulfobetaine methacrylate
SDS: Sodium dodecyl sulfate
CD: Cyclodextrin
CB: Cucurbituril
QCS-CD: Quaternized chitosan-graft-cyclodextrin
QCS-AD: Quaternized chitosan-graft-adamantane
GO-CD: Graphene oxide-graft-cyclodextrin
SF: Filamentin protein
SF-Chol: Filamentin protein grafted cholesterol
SF-CD: Filamentin protein grafted β-CD
GelMA: Gelatin methacrylate
AA: Adenine acrylate
Cu^2+^: Copper ion
BP: Bisphosphonate
CaP: Calcium phosphate
SIPN: Semi-interpenetrating polymer network
CEC: Carboxyethyl chitosan
OHA-AT: Oxidized hyaluronic acid-grafted aniline tetramer
DA: Diels–Alder
CNF: Cellulose nanofiber
MCAO: Middle cerebral artery occlusion
BMSC: Bone mesenchymal stem cell
TTC: 2,3,5-Triphenyltetrazolium chloride
DBS: Deep brain stimulation
TH: Tyrosine hydroxylase
GFAP: Glial fibrillary acidic protein
AD: Alzheimer's disease
AChE: Acetylcholinesterase
NMDA: *N*-Methyl-d-aspartate
BBB: Blood–brain barrier
TBI: Traumatic brain injury
HA-PBA: Phenylboronic acid grafted hyaluronic acid
Gel-Dopa: Dopamine-grafted gelatin
